# Impact of Adiponectin Overexpression on Allergic Airways Responses in Mice

**DOI:** 10.1155/2013/349520

**Published:** 2013-06-04

**Authors:** Norah G. Verbout, Leandro Benedito, Alison S. Williams, David I. Kasahara, Allison P. Wurmbrand, Huiqing Si, Andrew J. Halayko, Christopher Hug, Stephanie A. Shore

**Affiliations:** ^1^Department of Environmental Health, Harvard School of Public Health, 665 Huntington Avenue, Boston, MA 02115, USA; ^2^Departments of Physiology and Internal Medicine, University of Manitoba, Winnipeg, MB, Canada R3A 1R9; ^3^Children's Hospital, Boston, MA 02115, USA

## Abstract

Obesity is an important risk factor for asthma. Obese individuals have decreased circulating adiponectin, an adipose-derived hormone with anti-inflammatory properties. We hypothesized that transgenic overexpression of adiponectin would attenuate allergic airways inflammation and mucous hyperplasia in mice. To test this hypothesis, we used mice overexpressing adiponectin (Adipo Tg). Adipo Tg mice had marked increases in both serum adiponectin and bronchoalveolar lavage (BAL) fluid adiponectin. Both acute and chronic ovalbumin (OVA) sensitization and challenge protocols were used. In both protocols, OVA-induced increases in total BAL cells were attenuated in Adipo Tg versus WT mice. In the acute protocol, OVA-induced increases in several IL-13 dependent genes were attenuated in Adipo Tg versus WT mice, even though IL-13 per se was not affected. With chronic exposure, though OVA-induced increases in goblet cells numbers per millimeter of basement membrane were greater in Adipo Tg versus WT mice, mRNA abundance of mucous genes in lungs was not different. Also, adiponectin overexpression did not induce M2 polarization in alveolar macrophages. Our results indicate that adiponectin protects against allergen-induced inflammatory cell recruitment to the airspaces, but not development of goblet cell hyperplasia.

## 1. Introduction

Obesity is an important risk factor for asthma. Obesity increases the prevalence, the incidence, and possibly the severity of asthma, while weight loss improves many asthma outcomes in the obese [1, 2]. In addition, standard asthma therapeutic agents are less effective in obese versus lean asthmatics [3, 4]. 

Adiponectin is an adipocyte-derived hormone that declines in obesity [5]. Loss of adiponectin in obesity appears to have important functional consequences. In animal models, adiponectin deficiency exacerbates several obesity-related conditions, including insulin resistance and atherosclerosis [6, 7]. In obese human subjects, serum adiponectin levels are inversely correlated with the risk of type 2 diabetes, atherosclerosis, and hypertension [8–10]. Some, but not all, epidemiological studies suggest that obesity-related declines in adiponectin may also contribute to obesity-related asthma. For example, in a cohort of premenopausal women, the risk of asthma was greatest in subjects in the lowest tertile of serum adiponectin [11]. Similarly, in C57BL/6 mice, adiponectin deficiency worsens airway eosinophilia and macrophage recruitment induced by chronic allergen challenge [12]. In addition, we have shown that in lean Balb/C mice, continuous infusion of adiponectin via mini-Alzet pumps suppresses acute allergen-induced airway hyperresponsiveness, airway inflammation, and Th2 cytokine production [13]. Similarly, Ionescu et al. reported a reduction in allergic airway inflammation in allergen challenged mice treated intranasally with recombinant adiponectin [14]. These data suggest that manipulating adiponectin levels might have beneficial effects for asthma. However, none of these studies examined goblet cell metaplasia, a common feature of asthma (reviewed in [15]) and of animal models of asthma. Adiponectin receptors are expressed on airway epithelial cells [16], and we have previously reported that compared to wildtype (WT) mice, acute allergen sensitization and challenge results in reduced mucous cell staining in the airways of T-cadherin deficient mice, which have increased circulating adiponectin [17]. These data suggest that adiponectin may have the capacity to impact goblet cell metaplasia. 

Some of the observed beneficial effects of adiponectin in the airways [13, 14] may be related to its anti-inflammatory effects, particularly its effects on macrophages, although adiponectin also has proinflammatory effects [18–20]. Adiponectin decreases the ability of LPS or Toll-like receptor activation to elicit TNF*α* and IL-6 production from macrophages [21–24] and reduces eotaxin expression in bone marrow derived macrophages stimulated with TNF*α* and IL-4 [12]. Adiponectin also inhibits the transformation of macrophages to foam cells [25], which may explain the antiatherosclerotic effects of adiponectin. Cultured alveolar macrophages from adiponectin deficient mice demonstrate increased production of TNF*α*, both innately and upon stimulation with LPS [26]. Adiponectin has also been shown to promote an anti-inflammatory, M2 phenotype in macrophages of various origins [27–29]. Such effects may be magnified in the setting of allergic lung disease which is associated with induction of Th2 cytokines that also promote M2 polarization. 

The purpose of this study was to determine whether transgenic overexpression of adiponectin could affect allergic airways responses, including goblet cell hyperplasia. To do so, we used mice overexpressing adiponectin from a chicken beta-actin promoter/cytomegalovirus enhancer. We have previously reported that these mice have marked increases in serum adiponectin [30]. Two allergen sensitization and challenge protocols were employed. The first, an acute challenge protocol, was similar to that used in a previous study in which we examined the effects of adiponectin administered by mini-Alzet pumps [13] and was used as a proof of concept that adiponectin overexpressed in this manner would be effective. The second protocol, a chronic challenge protocol, results in allergic airways inflammation and goblet cell metaplasia, that is dependent on mast cells [31], as is allergic asthma in humans, whereas the acute protocol requires T cells but not mast cells [32]. The transgenic expression of adiponectin allowed increased levels of this adipokine to be present throughout the extended challenge period needed for this protocol.

## 2. Materials and Methods

### 2.1. Animals

This study was approved by the Harvard Medical Area Standing Committee on Animals. Transgenic mice overexpressing adiponectin (Adipo Tg) driven by a chicken beta-actin promoter with a cytomegalovirus enhancer and backcrossed onto a C57BL/6J background were generated as previously described [33]. Adipo Tg mice (which were ± for the transgene) were mated with WT littermates, and the Adipo Tg and littermate WT offspring of these matings were used in experiments described below. Primers used for genotyping were 5′-GCT TAT ATG TAT CGC TCA GCG TTC AG-3′ and 5′-CTT GTC GTC GTC GTC CTT GTA GTC-3′. Using these primers, a 438 bp product was observed in the Adipo Tg but not the WT mice.

### 2.2. Allergen Sensitization and Challenge

#### 2.2.1. Acute Protocol

Four-week-old male mice were sensitized to chicken egg albumin (ovalbumin (OVA), Grade V, Sigma-Aldrich Co., St. Louis, MO) on *day 1* by intraperitoneal injection of 20 *μ*g of OVA and adjuvant, 2 mg of aluminum hydroxide (Al(OH)_3_; J. T. Baker, Phillipsburg, NJ) dispersed in 0.2 mL of PBS. Mice were given a second injection of identical reagents on *day 14*. On *days 28* through *30*, mice were challenged for 30 min with an aerosol of either PBS containing 1% OVA or PBS alone, as previously described [34]. Mice were studied 24 h after the last OVA challenge.

#### 2.2.2. Chronic Protocol

Four-week-old male mice were sensitized to OVA on *days 1, 4, and 7* by intraperitoneal injection of 50 *μ*g OVA dispersed in 0.1 mL of PBS without alum. Beginning on *day 12*, mice were intranasally challenged once a week for 4 weeks with either sterile PBS or OVA (20 *μ*g in 30 *μ*L PBS) as described by others [31]. Mice were studied 24 h after the last OVA challenge.

### 2.3. Bronchoalveolar Lavage (BAL)

Mice were euthanized with an overdose of sodium pentobarbital, blood was drawn by cardiac puncture, and serum stored at −20°C for subsequent measurement of total serum adiponectin and IgE by ELISA. BAL was performed and the BAL cells and differentials counted as described previously [13, 34]. BAL supernatant was stored at −80°C and subsequently analyzed by ELISA for adiponectin, eotaxin, and IL-13 (R&D Systems Inc.). OVA specific IgE in serum was analyzed by ELISA (Cayman Chemical).

### 2.4. Histology and Morphometry

For mice in the chronic OVA protocol, twenty-four hours after the last exposure to OVA or PBS, mice were euthanized and lungs processed for histology as previously described [35]. Briefly, the lungs were inflated in situ at 20 cm of water with 4% paraformaldehyde and then removed en bloc. The left lung was cut, embedded in paraffin, and sections stained with periodic acid Schiff (PAS) to assess mucous-containing goblet cells. Sections were viewed using an upright light microscope equipped with SPOT CCD camera and Image Pro software (Olympus Canada, Mississauga, ON). Airways were selected for analysis that exhibited a maximum diameter: minimum diameter ratio that was less than ≤2 to avoid artifacts from airways cut obliquely. Basement membrane (BM) length was measured for all airways. For measurement of goblet cell abundance, a band-shaped region of interest extending from the BM to the edge of the epithelium was drawn, using a maximum length that avoided sectioning artifacts. Using Image Pro (Olympus) software, thresholds for detecting goblet cells were set to enable discrimination between individual mucous-positive cells, so that the numbers of cells per mm of BM could be determined. Images of at least 3 different airways were captured for each animal. Other sections were stained with hematoxylin and eosin to determine the inflammation index, a product of the severity and prevalence of inflammation, as described by Hamada et al. [36]. Severity was assigned a numerical value based upon the number of inflammatory cell infiltrate layers surrounding the airways or blood vessels (0, no cells; 1, ≤3 cell layers; 2, 4–9 cell layers; 3, 10 cell layers). Prevalence of inflammation was assigned a numerical value according to the percentage of airways or blood vessels in each section encompassed by inflammatory cells (0, none; 1, ≤25%; 2, 25–50%; 3, >50%). Medium to large sized airways were analyzed, and in each mouse, a minimum of 8 airways were examined. For both goblet cell hyperplasia and inflammation, a mean value for each mouse was computed and used for statistical analyses. 

### 2.5. RNA Extraction and Real-Time PCR

Lungs were harvested, frozen at −80°C, and subsequently homogenized for RNA extraction with RNeasy (Qiagen). Total RNA (1 *μ*g) was reverse transcribed using random hexamer primers and Superscript III (Invitrogen). Quantitative mRNA expression for each gene was performed using real-time PCR and SYBR Green (Applied Biosystems). Primers are described in Table 1. Values were calculated based on the ΔΔCT method, and 18S was used to control for total mRNA. For each set of primers, melting curve analysis yielded a single peak consistent with one PCR product.

### 2.6. Statistical Analysis of Results

Data were analyzed by factorial ANOVA (BAL and serum moieties, histological analyses, and lung mRNA expression) using STATISTICA software (StatSoft, Tulsa, OK). BAL cells were log transformed in order to conform to a normal distribution. Fisher's LSD test was used for post hoc comparisons. A *P* value < 0.05 was considered statistically significant.

## 3. Results

### 3.1. Acute Allergen Challenge Protocol

#### 3.1.1. Adiponectin Expression and Body Weight

**Figure 1:**
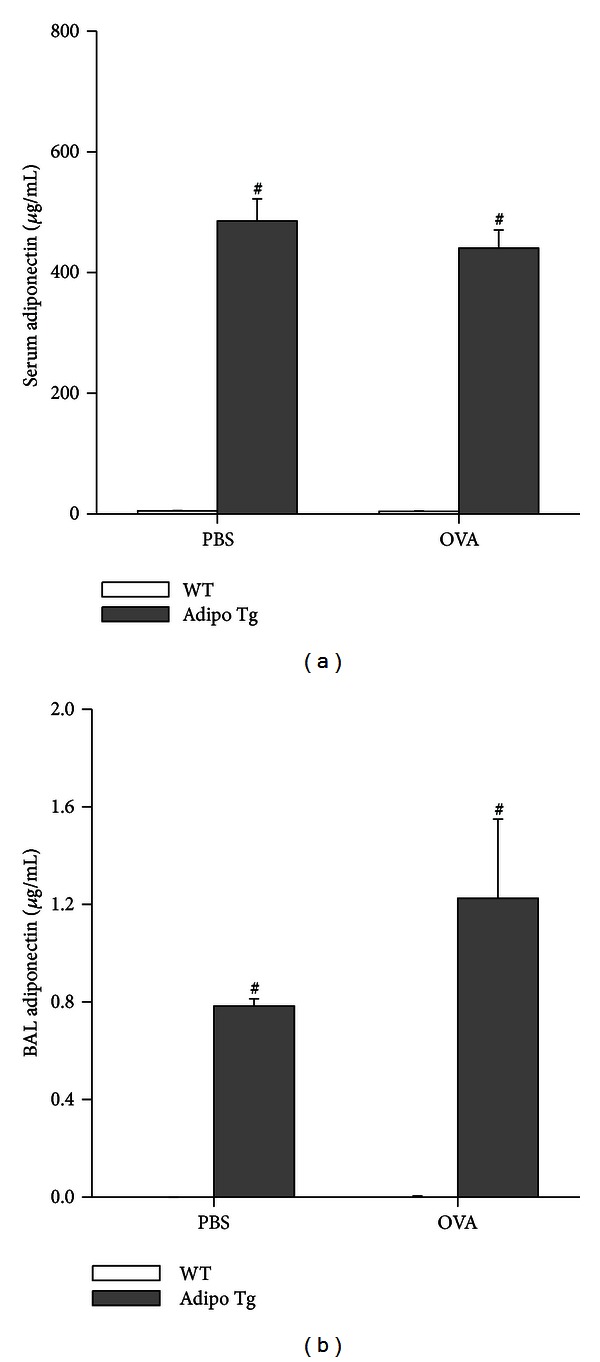
Serum (a) and bronchoalveolar lavage (BAL) fluid (b) adiponectin concentrations in wildtype (WT) and adiponectin transgenic (Adipo Tg) mice sensitized to ovalbumin (OVA) and challenged with aerosols of OVA or PBS every day for 3 days. Data shown are mean ± SEM of 5–7 mice per group. ^#^ *P* < 0.05 versus WT mice with the same exposure.

Compared to WT littermates, Adipo Tg mice had marked increases in both serum and BAL fluid adiponectin (Figure 1). Adiponectin levels in the serum were not significantly altered by exposure to OVA, though there was a trend towards increased BAL adiponectin in OVA versus PBS treated mice (*P* = 0.076). Neither adiponectin genotype nor OVA had any effect on body mass. Body mass averaged 26.9 ± 0.5, 26.7 ± 0.6, 26.5 ± 1.0, and 27.0 ± 0.7 g in WT mice challenged with PBS or OVA, and Adipo Tg mice challenged with PBS or OVA respectively.

#### 3.1.2. Pulmonary Inflammation

**Figure 2:**
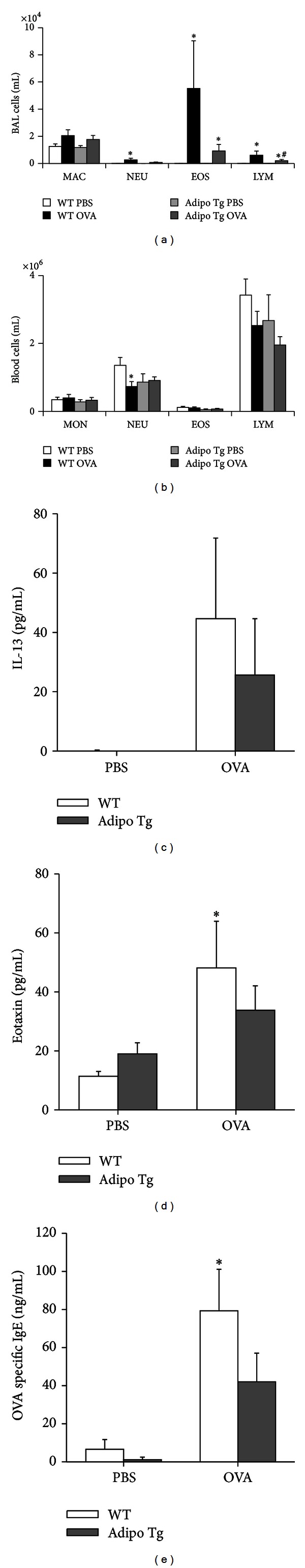
Leukocytes in BAL fluid (a) and blood (b) and BAL IL-13 (c), BAL eotaxin (d), and serum OVA-specific IgE (e) of wildtype (WT) and adiponectin transgenic (Adipo Tg) mice sensitized with ovalbumin (OVA) and exposed to aerosols of OVA or PBS. Data shown are mean ± SEM of 5-6 PBS and 9-10 OVA treated mice per group except for OVA-specific IgE (*n* = 5-6 for PBS and 12-13 for OVA). *P < 0.05 versus PBS challenged mice of the same genotype; ^#^ *P* < 0.05 versus WT mice with the same exposure. MAC: macrophages; NEU: neutrophils; EOS: eosinophils; LYM: lymphocytes; MON: mononuclear cells.

Factorial ANOVA indicated a significant effect of OVA versus PBS exposure on total BAL cells. Follow-up analysis indicated that the effect occurred in the WT mice. Total BAL cells increased from 2.7 ± 0.5 to 9.1 ± 4.2 × 10^4^ cells/mL in WT mice (*P* < 0.05), whereas there was no significant effect of OVA versus PBS exposure in Adipo Tg mice (2.1 ± 0.2 versus 3.9 ± 0.5 × 10^4^ cells/mL). OVA induced a significant increase in neutrophils, eosinophils, and lymphocytes (Figure 2(a)) in WT mice. Compared to WT mice, OVA-induced increases in lymphocytes were significantly reduced in adiponectin transgenic mice. A similar trend was observed for neutrophils and eosinophils but did not reach statistical significance. There was no effect of either OVA exposure or genotype on BAL epithelial cells (data not shown). In contrast, circulating leukocytes were not different between wildtype and adiponectin transgenic mice, except for a reduction in circulating neutrophils in WT OVA versus PBS treated mice (Figure 2(b)). These observations suggest that genotype-related reductions in infiltration of airspaces by inflammatory cells were not the result of a deficiency in the availability of these cells in the blood. 

Compared to PBS, OVA challenge increased BAL IL-13 (Figure 2(c)). There was no significant genotype-related difference, although there was a trend for reduced BAL IL-13 in Adipo Tg mice. OVA induced a significant increase in BAL eotaxin in WT but not Adipo Tg mice (Figure 2(d)). Similarly, OVA exposure increased OVA-specific serum IgE (Figure 2(e)) in WT mice, but not in Adipo Tg mice. 

#### 3.1.3. Pulmonary Gene Expression

**Figure 3:**
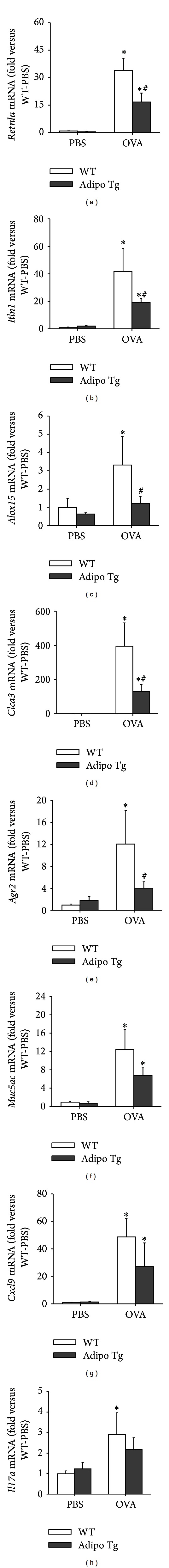
Pulmonary mRNA expression of *Retnla* (a), *Itln1* (b), *Alox15* (c), Clca3 (d), Agr2 (e), *Muc5ac* (f), *Cxcl9* (g), and *Il17a* (h) in wildtype (WT) and adiponectin transgenic (Adipo Tg) mice sensitized with ovalbumin (OVA) and exposed to aerosols of OVA or PBS every day for 3 days. Data shown are the means ± SEM of 5-6 mice/group. Data are normalized relative to 18S expression. *P < 0.05 versus PBS challenged mice of the same genotype; ^#^ *P* < 0.05 versus WT mice with the same challenge.

Real-time PCR was used to measure changes in mRNA abundance of genes known to be strongly induced in the lungs by OVA challenge in this protocol [37–44]. In WT mice, inhalation of OVA significantly increased pulmonary expression of *Retnla*, *Itln1*, *Alox15*, *Muc5ac*, *Cxcl9*, *Clca3, Agr2*, and *Il17a* mRNA (Figure 3). OVA-induced *Retnla*, *Itln1*, *Alox15, Clca3, and Agr2* mRNA abundance was significantly lower in Adipo Tg than WT mice. A similar trend was observed for *Muc5ac* and *Cxcl9,* but the effect did not reach statistical significance. *Il17a* mRNA abundance was not different in Adipo Tg and WT mice. 

### 3.2. Chronic Allergen Challenge Protocol

#### 3.2.1. Pulmonary Inflammation

**Figure 4:**
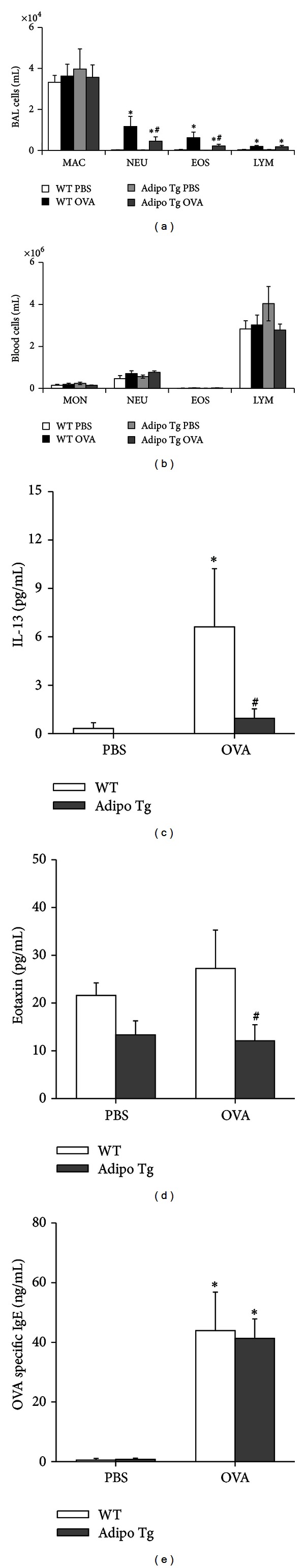
Leukocytes in BAL fluid (a) and blood (b), and BAL IL-13 (c), BAL eotaxin (d), and serum OVA-specific IgE (e) in wildtype (WT) and adiponectin transgenic (Adipo Tg) mice sensitized with ovalbumin (OVA) and challenged i.n. with OVA or PBS every week for 4 weeks. Data shown are mean ± SEM of 5–10 mice per group. *P < 0.05 versus PBS challenged mice of the same genotype; ^#^ *P* < 0.05 versus WT mice with the same challenge. MAC: macrophages; NEU: neutrophils; EOS: eosinophils; LYM: lymphocytes; MON: mononuclear cells.

Compared to PBS challenge, repeated intranasal OVA challenge caused significant increases in BAL neutrophils, eosinophils, and lymphocytes (Figure 4(a)), although eosinophils were lower than those induced with the acute challenge protocol (Figure 2(a)). Importantly, OVA-induced increases in BAL neutrophils and eosinophils were significantly attenuated in Adipo Tg versus WT mice. There was no effect of either OVA challenge or adiponectin genotype on circulating leukocytes (Figure 4(b)). Compared to PBS, OVA challenge increased BAL IL-13 in WT mice (Figure 4(c)). BAL IL-13 was significantly reduced in Adipo Tg versus WT OVA exposed mice. BAL eotaxin was also significantly lower in Adipo Tg versus WT OVA exposed mice (Figure 4(d)). Compared to PBS, OVA challenge caused a significant increase in serum OVA-specific IgE, but there was no effect of genotype (Figure 4(e)). 

**Figure 5:**
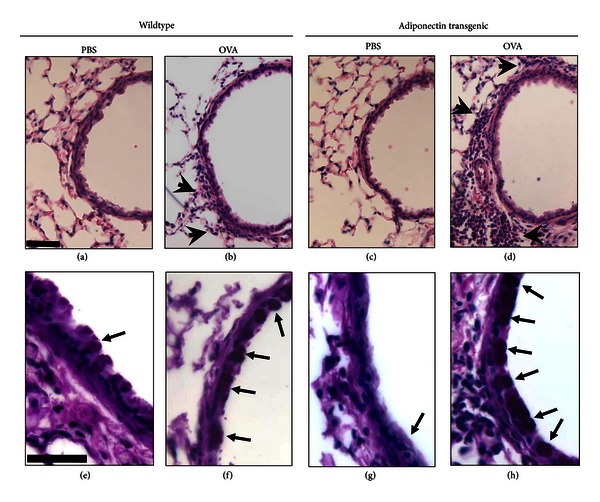
Histological sections of airways of wildtype (WT) and adiponectin transgenic (Adipo Tg) mice sensitized with ovalbumin (OVA) without alum and challenged intranasally with OVA or PBS every week for 4 weeks (chronic). Light micrographs showing H&E (panels (a)–(d)) or PAS (panels (e)–(h)) staining. Arrowheads in (a)–(d) indicate regions of inflammatory cell accumulation. Arrows in (e)–(h) indicate regions of “purple” staining that were recognized by Image Pro software (Olympus) during quantification of goblet cell abundance (see text). The bars in panels (a) and (e) = 10 *μ*m and correspond to all images in the upper and lower row, respectively. PAS staining for mucous-containing goblet cells ((e)–(h)) is from medium sized peripheral airways.

**Figure 6:**
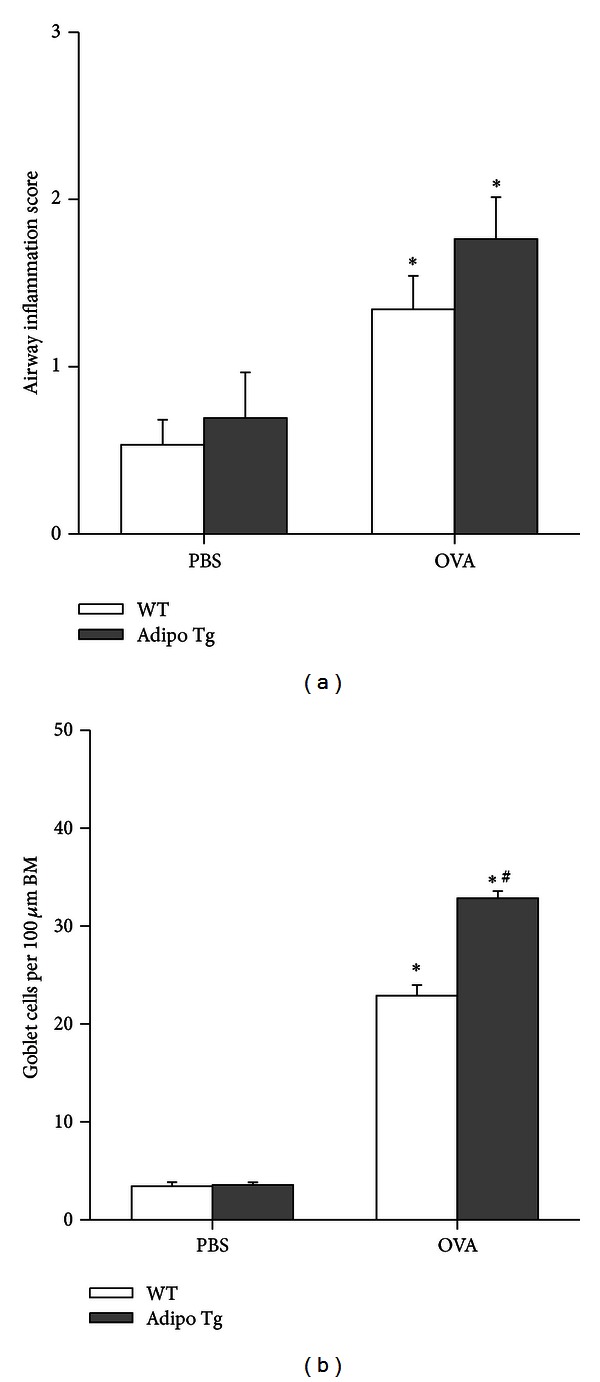
Quantitation of inflammatory cell accumulation around airways (a) and goblet cell metaplasia (b). Data shown are the means ± SEM of 4-5 mice/group in the PBS treated groups and 7 mice/group in the OVA treated groups. BM: basement membrane. *P < 0.05 versus PBS challenged mice of the same genotype; ^#^ *P* < 0.05 versus WT mice with the same challenge.

OVA challenge resulted in accumulation of inflammatory cells around airways (Figure 5), but there was no significant difference in the extent of these cell infiltrates in WT versus Adipo Tg mice (Figure 6(a)). We also examined goblet cell hyperplasia in these mice. Compared to PBS, chronic OVA i.n. challenge significantly increased the number of goblet cells per mm of BM in the airways in both WT and Adipo Tg mice (Figures 5 and 6(b)). However, there was a small but significant *increase* in goblet cell hyperplasia in the OVA challenged Adipo Tg versus WT mice. 

#### 3.2.2. Gene Expression

**Figure 7:**
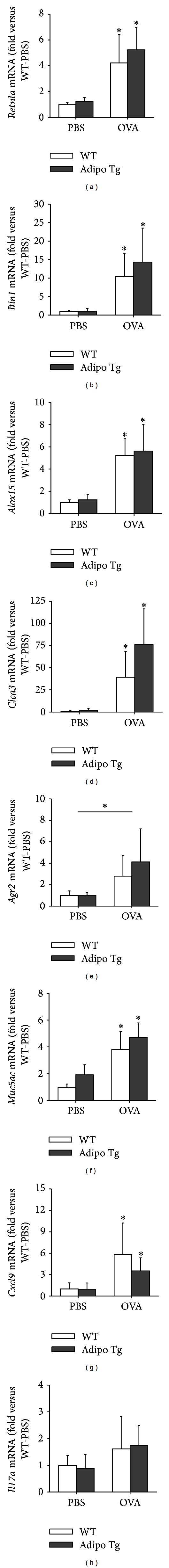
Pulmonary mRNA expression of *Retnla* (a), *Itln1* (b), *Alox15* (c), Clca3 (d), Agr2 (e), *Muc5ac* (f), *Cxcl9* (g), and *Il17a* (h) in wildtype (WT) and adiponectin transgenic (Adipo Tg) mice sensitized with ovalbumin (OVA) and challenged i.n. with OVA or PBS once a week for 4 weeks. Data shown are the means ± SEM of 6–11 mice/group. Data are normalized relative to 18S expression. *P < 0.05 versus PBS challenged mice of the same genotype.

Similar to the results obtained with the acute OVA exposure protocol, chronic intranasal instillation of OVA significantly increased pulmonary expression of *Retnla*, *Itln1*, *Alox15*, *Clca3, Agr2, Muc5ac*, and *Cxcl9* (Figure 7), although the magnitude of these changes was substantially reduced compared to the acute OVA protocol (Figure 3). However, in contrast to the acute OVA exposure protocol, OVA-induced increases in the expression of these genes were not significantly affected by transgenic status. *Il17a* mRNA expression was not induced by i.n. OVA (Figure 7(h)). 

**Figure 8:**
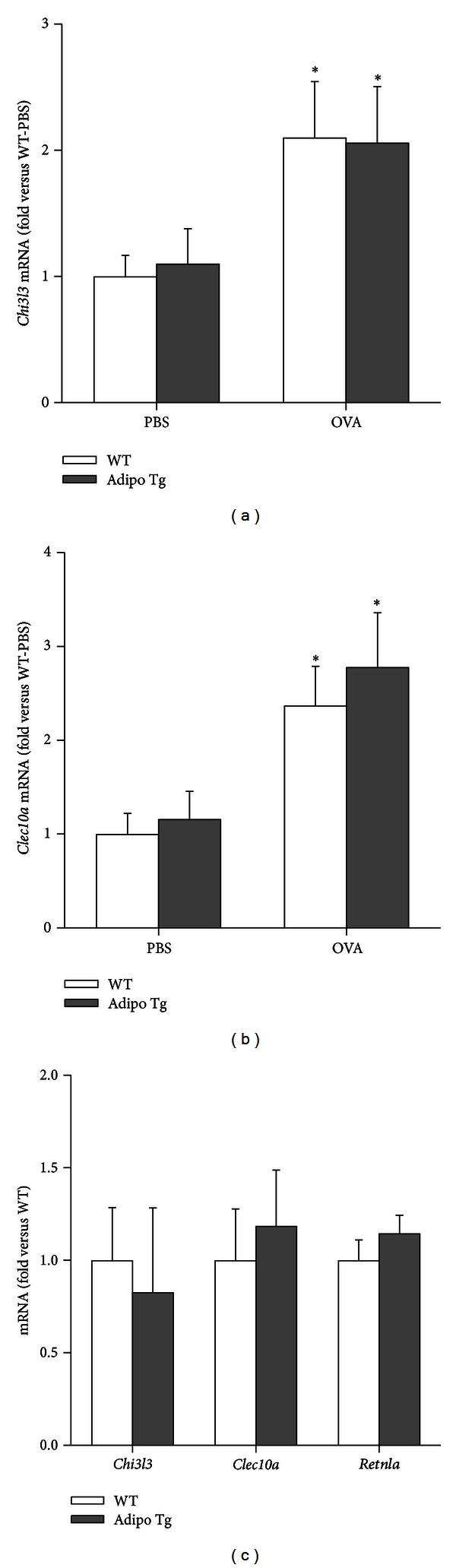
Pulmonary mRNA expression of *Chi3l3* (a) and *Clec10a* (b) in wildtype (WT) and adiponectin transgenic (Adipo Tg) mice sensitized with ovalbumin (OVA) and challenged i.n. with OVA or PBS. Data shown are the means ± SEM of 6–11 mice/group. *P < 0.05 versus PBS challenged mice of the same genotype. (c) Expression of *Chi3l3*, *Clec10a*, and *Retnla* mRNA in alveolar macrophages harvested from naïve wildtype and Adipo Tg mice. Results are mean ± SEM of data from 5–8 mice in each group. Data were normalized relative to 18S expression.

Since others have reported effects of adiponectin on macrophage phenotype [27, 29], we also measured additional markers of M2 phenotype in the lungs of these mice. Compared to PBS, chronic OVA challenge caused a significant increase in both *Chi3l3* and *Clec10a* (Mgl1), but there was no genotype-related difference in the induction of these genes by OVA (Figures 8(a) and 8(b)). We also evaluated expression of M2 associated genes in alveolar macrophages isolated from *naïve *WT and Adipo Tg mice (Figure 8(c)). *Chi3l3*, *Clec10a,* and *Retnla* mRNA levels were not different in macrophages isolated from WT and Adipo Tg mice. 

## 4. Discussion

Our data indicate that adiponectin overexpression results in a significant reduction in inflammatory cell recruitment to the airspaces (Figure 2) and in the induction of genes known to drive airway inflammation (Figure 3) after acute OVA challenge. A similar reduction in BAL inflammatory cells as well as reductions in BAL eotaxin and IL-13 with adiponectin overexpression was observed (Figure 4) when we used a chronic OVA challenge protocol in which inflammation is dependent on mast cells [31], a feature relevant to human allergic asthma. Importantly, others have previously reported that adiponectin deficiency results in increased allergic airways inflammation in this chronic challenge protocol, likely as a result of effects on chemokine expression, especially eotaxin [12]. Since mucous hypersecretion is largely refractory to current therapies, we also used the chronic protocol to determine whether adiponectin overexpression might also be effective against this aspect of the allergic airways phenotype. Interestingly, despite having no effect on Muc5ac mRNA abundance (Figure 7), we observed modestly higher goblet cell hyperplasia in mice with excess adiponectin (Figure 6). 

Adiponectin overexpression caused a reduction in the number of cells emigrating into the airspaces after acute OVA challenge, consistent with previous observations using exogenous adiponectin administration and a similar allergen challenge model [13]. We also observed significant changes in BAL eotaxin in WT but not Adipo Tg mice after acute OVA challenge. Eotaxin has been shown to contribute to eosinophil recruitment following allergen challenge in mice [45]. OVA specific IgE was also increased by OVA exposure in WT but not Adipo Tg mice (Figure 2(e)). These data confirm results of previous studies that examined the impact of exogenous adiponectin administration [13, 14], albeit in a different strain of mice (C57BL/6 versus Balb/c). Thus, our findings provided proof of concept that the transgenic mice would be a useful model to use for a chronic allergen challenge protocol in which elevated serum adiponectin needed to be sustained for an extended period (see below). 

Data from the acute challenge protocol extend results of previous studies by providing effects on expression of inflammatory genes relevant to allergic airways disease. For example, we observed a significant reduction in OVA-induced expression of *Retnla*, *Itln1*, *Clca3, Agr2,* and *Alox15* mRNA in Adipo Tg versus WT mice (Figure 3). Intelectin [37] and *Alox15* [42, 46] have each been shown to contribute to allergic airways inflammation in mice. Thus, our data suggest that adiponectin-dependent reductions in BAL cells may have been secondary to changes in the expression of these genes. In contrast to the effects of adiponectin overexpression on *Retnla*, *Itln1*, *Alox15, Clca3, and Agr2* mRNA (Figures 3(a)–3(e)), we did not observe any effects of adiponectin on induction of either *Cxcl9* or *Il17a* following acute OVA challenge (Figures 3(g) and 3(h)). Induction of *Retnla*, *Itln1*, *Alox15, Clca3,* and* Agr2* by acute OVA challenge is dependent upon IL-13, whereas induction of *Cxcl9* and *Il17a* is not [38, 42, 44, 46, 47]. Nevertheless, we did not observe any significant change in BAL IL-13 in Adipo Tg versus WT mice nor was there any difference in lung IL-13 mRNA abundance (data not shown). Taken together, the data suggest that adiponectin may mediate its effects on expression of *Retnla*, *Itln1*, *Alox15, Clca3,* and* Agr2* downstream of IL-13 rather than directly impacting IL-13 expression. 

We also examined the effects of adiponectin overexpression using a chronic OVA challenge protocol using weekly intranasal administration of OVA. We chose to examine this protocol because both inflammation and goblet cell hyperplasia require mast cells with this protocol [31], increasing the relevance to human asthma. In contrast, with the acute OVA challenge protocol, these outcomes are dependent upon T cells, but not mast cells [32]. Our data indicated that BAL inflammatory cells, IL-13, and eotaxin are reduced in Adipo Tg versus WT mice (Figure 4). These data are consistent with other reports showing that adiponectin deficiency increases allergic airways inflammation with this same challenge protocol [12]. Though our findings with respect to inflammation in chronic OVA-exposed mice (Figure 4) are similar to those from the acute OVA exposure mice (Figure 2), there were some differences. Whereas adiponectin overexpression reduced both inflammatory cell number and IL-13 in BAL in chronic OVA-exposed animals (Figure 4), we observed no difference in lung inflammatory gene expression (Figures 7 and 8). This contrasts with the marked reduction in gene expression induced by adiponectin overexpression with the acute OVA protocol (Figure 3). Consistent with the lack of effect on pulmonary gene expression in the chronic protocol, adiponectin overexpression had no effect on inflammation within the lung tissue: semi-quantitative analysis of airway inflammation from histological sections indicated no genotype-related effect (Figure 6). Taken together, the results suggest that in the chronic OVA model, adiponectin may act to prevent inflammatory/immune cell transit across the airway epithelium. 

We sought to determine whether transgenic expression of adiponectin might be effective in reducing the goblet cell metaplasia associated with allergic airways disease. Goblet cell metaplasia was induced in this chronic OVA challenge model (Figures 5 and 6), and there was also significant induction of genes involved in mucous cell metaplasia (*Muc5ac, Agr2, and Clca3*) (Figure 7). Nevertheless, we observed a modest but significant *increase* in goblet cells in Adipo Tg mice versus WT mice (Figures 5 and 6). To our knowledge, this is the first study examining effects of adiponectin on goblet cell metaplasia in the lungs, although others have reported significant reductions in other aspects of the remodeling process (pulmonary vascular and airway smooth muscle cell proliferation) in mice with elevated adiponectin [14, 48].

We were somewhat surprised to find that adiponectin overexpression augmented goblet cell hyperplasia, especially since IL-13, which is widely considered to drive this phenotype, was reduced in the Adipo Tg mice (Figure 4(c)). It is conceivable that the apparent increase in goblet cells in Adipo Tg mice was the result of less release of mucus into the airways and consequent increased retention of mucus within the cells themselves. However, the observation that expression of *Agr2* and* Clca3* also tended to increase in Adipo Tg versus WT mice (Figure 7) suggests otherwise. Of note, *Agr2* is an endoplasmic reticulum protein required for production of mucous glycoproteins like Muc5ac [49], while *Clca3* (gob-5) is required for OVA-induced goblet cell metaplasia and airspace inflammation in mice [50]. The increased goblet cell numbers and the trend towards increased Agr2 and Clca3 mRNA abundance in the Adipo Tg versus WT mice is consistent with the results of other investigators who have examined the impact of adiponectin on goblet cell hyperplasia in other organs [51, 52]. For example, Saxena et al. reported that adiponectin increases the differentiation of intestinal epithelial cells towards goblet cells [52]. Similarly, Li et al. reported that exogenous administration of adiponectin in the form of eye drops increased conjunctival goblet cell density in a mouse model of experimental dry eye [51].

In the Adipo Tg mice, adiponectin concentrations were extremely high both in the BAL fluid and the serum. It is possible that proinflammatory effects of adiponectin [18–20] impacted goblet cell numbers due to these sustained high concentrations. Contrary to this contention, we did observe reductions in both *Agr2* and *Clca3* mRNA in Adipo Tg versus WT mice upon acute OVA challenge. Others have used the chicken beta-actin promoter, cytomegalovirus enhancer that drove adiponectin expression in these mice to drive EGFP [53]. In the lungs of the resulting “green” mice, EGFP is mostly observed in the airway epithelium. Hence, it is conceivable that the enhanced goblet cell numbers are a consequence of coexpression of a transgene within these cells, although mRNA expression of epithelial genes (i.e., *Itln1, Retnla, Agr2, Clca3,* and *Muc5ac*) was not affected (Figure 7). Further investigation will be required to identify the cellular and molecular mechanisms at play. Nonetheless, our observations suggest that the molecular mechanisms controlling goblet cell hyperplasia in response to sustained chronic allergen challenge may be regulated by mechanisms that are different from those that regulate immune cell accumulation within the airways.

Expression of *Retnla*, *Itln1*, *Alox15, Agr2, and Clca3* was not reduced in Adipo Tg versus WT mice after chronic OVA challenge (Figure 7), whereas adiponectin overexpression caused a marked reduction in these same genes following acute OVA challenge (Figure 3). The differences in these observations may relate to differences in the mast cell/T cell dependence of the two models, as discussed above. For example, the cell targeted by adiponectin in acute protocol may be T cells themselves. It is also important to note that the magnitude of the inflammation induced by chronic OVA challenge was substantially less than that induced by acute challenge (BAL eosinophilia in Figures 2 and 4, and gene expression in Figures 3 and 7). It is conceivable that with the more subdued inflammation in the chronic protocol, endogenous levels of adiponectin are sufficient to provide maximal inhibition of gene expression, and increasing adiponectin further has no effect. It is also possible that differences in the magnitude of inflammation induced in the two models contribute directly to differences in gene expression, since RNA was evaluated in whole lung: differences in lung gene expression may reflect the different cell populations in the lungs under the different conditions and the different gene expression profiles of these cells.

 Our data do not support a role for adiponectin in alternative activation of *lung* macrophages. Firstly, we observed no difference in the expression of genes classically associated with M2 status in alveolar macrophages harvested from naïve Adipo Tg versus WT mice (Figure 8(c)), despite evidence that these macrophages normally exist in a microenvironment replete with excess adiponectin (Figure 1(b)). Secondly, adiponectin overexpression either had no effect on (Figures 7 and 8) or significantly reduced (Figure 3) OVA-induced increases in the expression of *Retnla*, *Chi3l3*, and *Clec10a*. In contrast, in macrophages from other sources, including peritoneal macrophages, adipose tissue macrophages, Kupffer cells, bone marrow or monocyte-derived macrophages, and macrophage cell lines, adiponectin augments the induction of these M2 genes either on its own or in conjunction with Th2 cytokines [27–29]. Effects of adiponectin on expression of M2 genes in lung macrophages has not previously been examined, although there is evidence that adiponectin deficiency increases M1 type activation of lung macrophages, as indicated by increased MHC class 2 expression and augmented TNF*α* production [26, 27]. However, adiponectin can also have proinflammatory effects on lung macrophages, since macrophages from adiponectin deficient mice produce less TNF*α* in response to cigarette smoke extract than wildtype mice [54].

## 5. Conclusions

In conclusion, transgenic overexpression of adiponectin in mice protected against the development of allergic airways inflammation and expression of IL-13 dependent genes after acute OVA challenge. Airspace inflammation was also reduced by adiponectin overexpression after chronic OVA. In contrast, inflammatory gene expression was not impacted by adiponectin overexpression, and goblet cell hyperplasia was increased. The data indicate that the ability of adiponectin supplementation to ameliorate allergic airways disease is dependent on the nature of the model employed. From a clinical perspective, our data suggest that increasing circulating adiponectin in obese individuals or agonists that target adiponectin receptors would be expected to decrease allergic airway inflammation but would not be beneficial in reducing the mucous metaplasia characteristic of allergic asthma. 

## Figures and Tables

**Table 1:** Primers used for qRT-PCR.

Gene	Forward primer (5′ → 3′)	Reverse primer (5′ → 3′)
*Alox15 *	ATGGTGCTGAAGCGGTCTAC	ATCCGCTTCAAACAGAGTGC
*Cxcl9 *	TCCTCTTGGGCATCATCTTC	TGAGGGATTTGTAGTGGATCG
*Il13 *	CAGCATGGTATGGAGTGTGG	AGGCCATGCAATATCCTCTG
*Il17a *	CTCCAGAAGGCCCTCAGACTA	AGCTTTCCCTCCGCATTGACACAG
*Itln1 *	AGAACCTGGACACCAACAGG	GTGCGCAGGAAATAGAGACC
*Clec10a *	CAGTGGAGAAGAGGGAGCAG	AGCCGTTGTTCTTGAGGTTG
*Muc5ac *	ACCCAGAGGGTCAGTGTGAG	TTGGGATAGCATCCTTCCAG
*Retnla *	TCGTGGAGAATAAGGTCAAGG	GGAGGCCCATCTGTTCATAG
*Chi3l3 *	GAAGGAGCCACTGAGGTCTG	TTGTTGTCCTTGAGCCACTG
*Clca3 *	GATTCCCGAGAGCTGGAAG	TGTTCGGTGTAGGGCTCATC
*Agr2 *	GCGATCAGCTCATCTGGACT	GCTTGACTGTGTGGGCATT
*18S ribosomal RNA *	GTAACCCGTTGAACCCCATT	CCATCCAATCGGTAGTAGCG

## References

[1] Ford ES (2005). The epidemiology of obesity and asthma. *Journal of Allergy and Clinical Immunology*.

[2] Taylor B, Mannino D, Brown C, Crocker D, Twum-Baah N, Holguin F (2008). Body mass index and asthma severity in the National Asthma Survey. *Thorax*.

[3] Lavoie KL, Bacon SL, Labrecque M, Cartier A, Ditto B (2006). Higher BMI is associated with worse asthma control and quality of life but not asthma severity. *Respiratory Medicine*.

[4] Saint-Pierre P, Bourdin A, Chanez P, Daures JP, Godard P (2006). Are overweight asthmatics more difficult to control?. *Allergy*.

[5] Kadowaki T, Yamauchi T, Kubota N (2008). The physiological and pathophysiological role of adiponectin and adiponectin receptors in the peripheral tissues and CNS. *FEBS Letters*.

[6] Kubota N, Terauchi Y, Yamauchi T (2002). Disruption of adiponectin causes insulin resistance and neointimal formation. *Journal of Biological Chemistry*.

[7] Nawrocki AR, Rajala MW, Tomas E (2006). Mice lacking adiponectin show decreased hepatic insulin sensitivity and reduced responsiveness to peroxisome proliferator-activated receptor *γ* agonists. *Journal of Biological Chemistry*.

[8] Hotta K, Funahashi T, Arita Y (2000). Plasma concentrations of a novel, adipose-specific protein, adiponectin, in type 2 diabetic patients. *Arteriosclerosis, Thrombosis, and Vascular Biology*.

[9] Patel DA, Srinivasan SR, Xu JH, Chen W, Berenson GS (2006). Adiponectin and its correlates of cardiovascular risk in young adults: the Bogalusa Heart Study. *Metabolism*.

[10] Shargorodsky M, Boaz M, Goldberg Y (2009). Adiponectin and vascular properties in obese patients: is it a novel biomarker of early atherosclerosis. *International Journal of Obesity*.

[11] Sood A, Cui X, Quails C (2008). Association between asthma and serum adiponectin concentration in women. *Thorax*.

[12] Medoff BD, Okamoto Y, Leyton P (2009). Adiponectin deficiency increases allergic airway inflammation and pulmonary vascular remodeling. *The American Journal of Respiratory Cell and Molecular Biology*.

[13] Shore SA, Terry RD, Flynt L, Xu A, Hug C (2006). Adiponectin attenuates allergen-induced airway inflammation and hyperresponsiveness in mice. *Journal of Allergy and Clinical Immunology*.

[14] Ionescu LI, Alphonse RS, Arizmendi N (2012). Airway delivery of soluble factors from plastic-adherent bone marrow cells prevents murine asthma. *The American Journal of Respiratory Cell and Molecular Biology*.

[15] Siddiqui S, Martin JG (2008). Structural aspects of airway remodeling in asthma. *Current Allergy and Asthma Reports*.

[16] Miller M, Cho JY, Pham A, Ramsdell J, Broide DH (2009). Adiponectin and functional adiponectin receptor 1 are expressed by airway epithelial cells in chronic obstructive pulmonary disease. *Journal of Immunology*.

[17] Williams AS, Kasahara DI, Verbout NG (2012). Role of the adiponectin binding protein, T-cadherin (Cdh13), in allergic airways responses in mice. *PLoS One*.

[18] Haugen F, Drevon CA (2007). Activation of nuclear factor-*κ*B by high molecular weight and globular adiponectin. *Endocrinology*.

[19] Mcdonald EA, Wolfe MW (2011). The pro-inflammatory role of adiponectin at the maternal-fetal interface. *The American Journal of Reproductive Immunology*.

[20] Kitahara K, Kusunoki N, Kakiuchi T, Suguro T, Kawai S (2009). Adiponectin stimulates IL-8 production by rheumatoid synovial fibroblasts. *Biochemical and Biophysical Research Communications*.

[21] Furukawa K, Hori M, Ouchi N (2004). Adiponectin down-regulates acyl-coenzyme A: cholesterol acyltransferase-1 in cultured human monocyte-derived macrophages. *Biochemical and Biophysical Research Communications*.

[22] Ouchi N, Kihara S, Arita Y (2001). Adipocyte-derived plasma protein, adiponectin, suppresses lipid accumulation and class A scavenger receptor expression in human monocyte-derived macrophages. *Circulation*.

[23] Yokota T, Oritani K, Takahashi I (2000). Adiponectin, a new member of the family of soluble defense collagens, negatively regulates the growth of myelomonocytic progenitors and the functions of macrophages. *Blood*.

[24] Masaki T, Chiba S, Tatsukawa H (2004). Adiponectin protects LPS-induced liver injury through modulation of TNF-*α* in KK-Ay obese mice. *Hepatology*.

[25] Tian L, Luo N, Klein RL, Chung BH, Garvey WT, Fu Y (2009). Adiponectin reduces lipid accumulation in macrophage foam cells. *Atherosclerosis*.

[26] Summer R, Little FF, Ouchi N (2008). Alveolar macrophage activation and an emphysema-like phenotype in adiponectin-deficient mice. *The American Journal of Physiology*.

[27] Ohashi K, Parker JL, Ouchi N (2010). Adiponectin promotes macrophage polarization toward an anti-inflammatory phenotype. *Journal of Biological Chemistry*.

[28] Mandal P, Pratt BT, Barnes M, McMullen MR, Nagy LE (2011). Molecular mechanism for adiponectin-dependent m2 macrophage polarization link between the metabolic and innate immune activity of full-length adiponectin. *Journal of Biological Chemistry*.

[29] Lovren F, Pan Y, Quan A (2010). Adiponectin primes human monocytes into alternative anti-inflammatory M2 macrophages. *The American Journal of Physiology*.

[30] Zhu M, Hug C, Kasahara DI (2010). Impact of adiponectin deficiency on pulmonary responses to acute ozone exposure in mice. *The American Journal of Respiratory Cell and Molecular Biology*.

[31] Yu M, Tsai M, Tam SY, Jones C, Zehnder J, Galli SJ (2006). Mast cells can promote the development of multiple features of chronic asthma in mice. *Journal of Clinical Investigation*.

[32] Takeda K, Hamelmann E, Joetham A (1997). Development of eosinophilic airway inflammation and airway hyperresponsiveness in mast cell-deficient mice. *Journal of Experimental Medicine*.

[33] Wong GW, Krawczyk SA, Kitidis-Mitrokostas C (2009). Identification and characterization of CTRP9, a novel secreted glycoprotein, from adipose tissue that reduces serum glucose in mice and forms heterotrimers with adiponectin. *FASEB Journal*.

[34] Johnston RA, Zhu M, Rivera-Sanchez YM (2007). Allergic airway responses in obese mice. *The American Journal of Respiratory and Critical Care Medicine*.

[35] Zhu M, Liu PY, Kasahara DI (2011). Role of Rho kinase isoforms in murine allergic airway responses. *European Respiratory Journal*.

[36] Hamada K, Suzaki Y, Goldman A (2003). Allergen-independent maternal transmission of asthma susceptibility. *Journal of Immunology*.

[37] Gu N, Kang G, Jin C (2010). Intelectin is required for IL-13-induced monocyte chemotactic protein-1 and -3 expression in lung epithelial cells and promotes allergic airway inflammation. *The American Journal of Physiology*.

[38] Kuperman DA, Lewis CC, Woodruff PG (2005). Dissecting asthma using focused transgenic modeling and functional genomics. *Journal of Allergy and Clinical Immunology*.

[39] Schnyder-Candrian S, Togbe D, Couillin I (2006). Interleukin-17 is a negative regulator of established allergic asthma. *Journal of Experimental Medicine*.

[40] Zhang L, Wang M, Kang X (2009). Oxidative stress and asthma: proteome analysis of chitinase-like proteins and FIZZ1 in lung tissue and bronchoalveolar lavage fluid. *Journal of Proteome Research*.

[41] Alimam MZ, Piazza FM, Selby DM, Letwin N, Huang L, Rose MC (2000). Muc-5/5ac mucin messenger RNA and protein expression is a marker of goblet cell metaplasia in murine airways. *The American Journal of Respiratory Cell and Molecular Biology*.

[42] Andersson CK, Claesson HE, Rydell-Törmänen K, Swedmark S, Hällgren A, Erjefält JS (2008). Mice lacking 12/15-lipoxygenase have attenuated airway allergic inflammation and remodeling. *The American Journal of Respiratory Cell and Molecular Biology*.

[43] Di Valentin E, Crahay C, Garbacki N (2009). New asthma biomarkers: lessons from murine models of acute and chronic asthma. *The American Journal of Physiology*.

[44] Lewis CC, Aronow B, Hutton J (2009). Unique and overlapping gene expression patterns driven by IL-4 and IL-13 in the mouse lung. *Journal of Allergy and Clinical Immunology*.

[45] Rothenberg ME, MacLean JA, Pearlman E, Luster AD, Leder P (1997). Targeted disruption of the chemokine eotaxin partially reduces antigen-induced tissue eosinophilia. *Journal of Experimental Medicine*.

[46] Lindley AR, Crapster-Pregont M, Liu Y, Kuperman DA (2010). 12/15-lipoxygenase is an interleukin-13 and interferon-*γ* counterregulated-mediator of allergic airway inflammation. *Mediators of Inflammation*.

[47] Thai P, Chen Y, Dolganov G, Wu R (2005). Differential regulation of MUC5AC/Muc5ac and hCLCA-1/mGob 5 expression in airway epithelium. *The American Journal of Respiratory Cell and Molecular Biology*.

[48] Weng M, Raher MJ, Leyton P (2011). Adiponectin decreases pulmonary arterial remodeling in murine models of pulmonary hypertension. *The American Journal of Respiratory Cell and Molecular Biology*.

[49] Schroeder BW, Verhaeghe C, Park SW (2012). AGR2 is induced in asthma and promotes allergen-induced mucin overproduction. *The American Journal of Respiratory Cell and Molecular Biology*.

[50] Long AJ, Sypek JP, Askew R (2006). Gob-5 contributes to goblet cell hyperplasia and modulates pulmonary tissue inflammation. *The American Journal of Respiratory Cell and Molecular Biology*.

[51] Li Z, Woo JM, Chung SW (2013). Therapeutic effect of topical adiponectin in a mouse model of desiccating stress-induced dry eye. *Investigative Ophthalmology and Visual Science*.

[52] Saxena A, Baliga MS, Ponemone V Mucus and adiponectin deficiency: role in chronic inflammation-induced colon cancer.

[53] Ma DF, Tezuka H, Kondo T (2010). Differential tissue expression of enhanced green fluorescent protein in `green mice’. *Histology and histopathology*.

[54] Miller M, Pham A, Cho JY, Rosenthal P, Broide DH (2010). Adiponectin-deficient mice are protected against tobacco-induced inflammation and increased emphysema. *The American Journal of Physiology*.

